# Assessment of free fatty acids and cholesteryl esters delivered in liposomes as novel class of antibiotic

**DOI:** 10.1186/s13104-016-2138-8

**Published:** 2016-07-08

**Authors:** Annie H. Cheung Lam, Natalie Sandoval, Ritambhara Wadhwa, Janine Gilkes, Thai Q. Do, William Ernst, Su-Ming Chiang, Suzanne Kosina, H. Howard Xu, Gary Fujii, Edith Porter

**Affiliations:** Department of Biological Sciences, California State University Los Angeles, 5151 State University Drive, Los Angeles, CA 90032 USA; Molecular Express, Inc., Rancho Dominguez, CA USA

**Keywords:** Antibiotic, Antimicrobial lipids, Drug delivery, Liposomes, Multidrug-resistance, HAI, Innate immunity

## Abstract

**Background:**

Healthcare associated infections (HAI) with multidrug-resistant (MDR) bacteria continue to be a global threat, highlighting an urgent need for novel antibiotics. In this study, we assessed the potential of free fatty acids and cholesteryl esters that form part of the innate host defense as novel antibacterial agents for use against MDR bacteria.

**Methods:**

Liposomes of six different phospholipid mixtures were employed as carrier for six different fatty acids and four different cholesteryl esters. Using a modified MIC assay based on DNA quantification with the fluoroprobe Syto9, formulations were tested against Gram-positive and Gram-negative bacteria implicated in HAI. Formulations with MIC values in the low μg/mL range were further subjected to determination of minimal bactericidal activity, hemolysis assay with sheep erythrocytes, and cytotoxicity testing with the human liver cell line HepG2. The potential for synergistic activity with a standard antibiotic was also probed.

**Results:**

Palmitic acid and stearic acid prepared in carrier 4 (PA4 and SA4, respectively) were identified as most active lipids (MIC against MDR *Staphylococcus epidermidis* was 0.5 and 0.25 μg/mL, respectively; MIC against vancomycin resistant *Enterococcus faecalis* (VRE) was 2 and 0.5 μg/mL, respectively). Cholesteryl linoleate formulated with carrier 3 (CL3) exhibited activity against the *S. epidermidis* strain (MIC 1 μg/mL) and a *Pseudomonas aeruginosa* strain (MIC 8 μg/mL) and lowered the vancomycin MIC for VRE from 32–64 μg/mL to as low as 4 μg/mL. At 90 μg/mL PA4, SA4, and CL3 effected less than 5 % hemolysis over 3 h and PA4 and CL3 did not exhibit significant cytotoxic activity against HepG2 cells when applied at 100 μg/mL over 48 h.

**Conclusions:**

Our results showed that selected fatty acids and cholesteryl esters packaged with phospholipids exhibit antibacterial activity against Gram-positive and Gram-negative bacteria and may augment the activity of antibiotics. Bactericidal activity could be unlinked from hemolytic and cytotoxic activity and the type of phospholipid carrier greatly influenced the activity. Thus, fatty acids and cholesteryl esters packaged in liposomes may have potential as novel lipophilic antimicrobial agents.

**Electronic supplementary material:**

The online version of this article (doi:10.1186/s13104-016-2138-8) contains supplementary material, which is available to authorized users.

## Background

Healthcare associated infections (HAI) are one of the leading causes of preventable deaths in the US, and present a significant economic burden in healthcare costs. Warnings regarding infectious agents developing into multidrug-resistant forms and the possibility of future pandemic outbreaks have been repeatedly issued by the Centers for Disease Control and Prevention and the World Health Organization. The global dissemination of drug resistant carbapenemase-producing Enterobacteriaceae in the healthcare setting [1] is just one of the more recent examples. Other causes of HAI with multidrug-resistant bacteria include methicillin-resistant *Staphylococcus aureus* (MRSA), vancomycin-resistant *Enterococcus faecalis* (VRE), *Pseudomonas aeruginosa*, and *Acinetobacter baumannii* [2]. Multidrug-resistant *Staphylococcus epidermidis* has become notorious for catheter associated infections. Nationwide, at least one case of HAI occurs for every 25 patients [3]. Despite some progress in containing the occurrence of HAI through infection control measures [4], an urgent need for the development of novel antibacterial agents remains. No new class of antibiotic has been discovered since 1987 and the last six approved new drugs are all modifications of existing drugs [5, 6]. Drug discovery through target-focused screening of large libraries of synthetic compounds has failed with a major reason being insufficient compound penetration into the microbial cell [7], and transition of natural antimicrobial peptides to the clinical market has proven to be difficult [8, 9]. Ongoing efforts include antisense RNA targeting essential microbial genes [10] and exploitations of uncultured bacteria [11]. However, host derived lipids, increasingly recognized as important antimicrobial effectors of the innate host defense [12, 13], might also be exploited for novel drug design.

Lipids are a widely heterogeneous group of molecules that share hydrophobic or mixed hydrophobic/hydrophilic properties [14]. The major lipid classes include: fatty acids- the basic building blocks for more complex lipids, cholesteryl esters, and phospholipids. Cholesteryl esters are formed through esterification of a fatty acid to cholesterol. Phospholipids typically consist of a glycerol with two fatty acid residues attached, a phosphate group and additions such as choline, an alcohol, or amines. The antimicrobial activity of fatty acids has been known for some time (reviewed in [15]) while antimicrobial properties of cholesteryl esters have been more recently discovered [16].

Motivated by the urgent need for novel antibacterial strategies, the main objective for this study was to examine whether lipids identified as antibacterial effectors in body fluids may have a potential to be used in innovative drug design. Due to their inherent hydrophobicity, lipids require carriers in aqueous environments. Liposomes have been used in the past for delivery of compounds with hydrophobic properties [17] offering in some cases the benefit of reducing cytotoxicity [18]. Liposomes are phospholipid vesicles where the polar head groups of phospholipids interact with the aqueous outer environment and the fatty acid tails provide a hydrophobic environment for lipophilic compounds. When used as carrier, liposomes also provide the advantage of allowing incorporation of tags for targeted delivery [19, 20].

## Methods

### Bacterial strains

The following strains were purchased from American Type Culture Collection (ATCC, Manassas, VA, multidrug-resistant *S. epidermidis* ATCC 700566, methicillin resistant *S. aureus* ATCC 33591 (MRSA), vancomycin resistant *E. faecalis* ATCC 700802 (VRE), *E. cloacae* ATCC 49141, *P. aeruginosa* ATCC 9027, and *A. baumanii* ATCC 19606. In addition, a clinical cystic fibrosis isolate of *P. aeruginosa* (PACF, Welsh laboratory, University of Iowa; also described in [16]) was used. Stock cultures were stored at −80 °C using the CryoSaver system (Hardy Diagnostics, Santa Maria, CA). For each assay, isolated colonies subcultured from freshly thawed bacteria were used to inoculate the ATCC recommended broth media purchased from Becton, Dickinson, and Company (BD, Franklin Lakes, NJ; nutrient broth for MRSA and *A. baumannii*, brain heart infusion for VRE, tryptic soy broth for the remainder). After overnight culture at 37 °C (20–24 h for *A. baumannii*, 16–18 h for others) bacteria were adjusted to McFarland 0.5 in 140 mM NaCl, diluted 100-fold, and inoculated into 1.1-fold concentrated cation-adjusted Mueller–Hinton broth (MHB+ , Teknova, Hollister, CA) to yield 5 × 10^5^ CFU/mL.

### Antibiotics

Oxacillin, cefotaxime, vancomycin, tetracycline, and ciprofloxacin were purchased from Sigma-Aldrich in salt form, dissolved in dH_2_O, and kept as stock solution (10 mg/mL) at −20 °C.

### Lipids

Lipids investigated (test lipids, Table 1) included free fatty acids (palmitic, stearic, oleic, linoleic, arachidonic, and docosahexaenoic acid) and cholesteryl esters (cholesteryl palmitate, -oleate, -linoleate, and -arachidonate) and were purchased from Sigma-Aldrich, St. Louis, MO. Carrier lipids included phospholipids and cholesterol. The phospholipids 1,2-dimyristoyl-sn-glycero-3-phosphatidylcholine (DMPC), 1,2-dipalmitoyl-sn-glycero-3-phosphocholine (DPPC), 1,2-distearoyl-sn-glycero-3-phosphatidyl-(10-rac-glycerol) (DSPG), and Hydrogenated Soy Phosphatidylcholine (HSPC; SPC-3) were purchased from Lipoid GmbH, Ludwigshafen, Germany. The phospholipid 1,2-dimyristoyl-sn-glycero-3-phosphatidyl-(10-rac-glycerol) (DMPG) was purchased from Nippon Fine Chemical, Osaka, Japan, and cholesterol from NOF Corporation, Tokyo, Japan.

**Table 1:** Lipids investigated in this study

Lipid class	Test lipid	Code	Molecular formula	Molecular weight^a^	Double bonds^b^
Free fatty acids	Palmitic acid	PA	C_16_H_32_O_2_	256.42	0
	Stearic acid	SA	C_18_H_36_O_2_	284.48	0
	Oleic acid	OA	C_18_H_34_O_2_	282.46	1
	Linoleic acid	LA	C_18_H_32_O_2_	280.45	2
	Arachidonic acid	AA	C_20_H_32_O_2_	304.47	4
	Docosahexaenoic acid	DA	C_22_H_32_O_2_	328.49	6
Cholesteryl esters	Cholesteryl palmitate	CP	C_43_H_76_O_2_	625.06	0
	Cholesteryl oleate	CO	C_45_H_78_O_2_	651.10	1
	Cholesteryl linoleate	CL	C_45_H_76_O_2_	649.08	2
	Cholesteryl arachidonate	CA	C_47_H_76_O_2_	673.11	4

^a^As provided by the manufacturer

^b^Number of unsaturated double bonds in the free or esterified fatty acid

### Liposome preparation

Six different carrier liposome formulations (carrier-1–6) were generated using the phospholipids and cholesterol listed above (carrier 1: HSPC; carrier 2: DPPC; carrier 3: DMPC; carrier 4: HSPC:DSPG [0.75:0.5]; carrier 5: DPPC:cholesterol [0.8:0.05]; carrier 6: HSPC:cholesterol [0.70:0.10], all in molar ratios). Principles of the liposome preparation have been described more in detail earlier [21]. Liposomes were prepared by dissolving the carrier and test lipids (0.8:0.2, respectively) in chloroform/methanol (1:1 *v/v*). The organic solvents were evaporated under a stream of nitrogen gas at 65 °C, and residual solvent removed in vacuo for >24 h. Unilamellar liposomes were formed by hydrating the lipid films with 9 % sucrose and probe sonicating until translucent. The liposomes were then sterilized by passage through a 0.22 µm filter. Each liposome preparation was characterized by dynamic light scattering (UPA 150, Microtrac, Montgomeryville, PA; see Additional file 1: Table S1). Liposomes were stored in 9 % sucrose at 1 mg/mL test lipid (5–10 mg total lipid/mL) for up to 6 month at 4 °C. As a control 9 % sucrose was used. When necessary, liposomal formulations containing test lipids were diluted with 9 % sucrose to ten-fold concentrated working stocks. Stock solutions for the respective carrier liposomes without test lipids were diluted so that the phospholipid concentration was the same as the phospholipid concentration in the formulations with test lipid.

### Determination of minimal inhibitory concentration (MIC)

Following Clinical and Laboratory Standards Institute M07-A9 for broth microdilution assay [22], 10 μL aliquots of serially diluted 10-fold concentrated antibiotic were added to general assay 96 well- round bottom polystyrene microtiter plates followed by the addition of 90 μL of diluted bacteria as prepared in above. For sterility control, 1.1-fold cation-adjusted Mueller–Hinton broth (MHB+) without bacteria was included in the testing. The plates were then incubated at 37 °C for 16–24 h, as recommended, in air and turbidity was read at 650 nm. MIC was the lowest concentration of antibiotic that prevented growth reflected in measurable turbidity.

### Modified minimal inhibitory concentration assay

Due to the inherent turbidity of the liposomal formulations interfering with absorbance readings, the MIC protocol was modified; after completion of the MIC assay the DNA content was quantified with the fluoroprobe Syto9 (Invitrogen, Carlsbad, CA). Syto9 freely crosses cell membranes and binds to DNA upon which green fluorescence is emitted. The degree of fluorescence correlates with the amount of DNA, thus with the bacterial concentration in a sample, regardless of sample turbidity contributed by the liposomal formulations [16]. Using black untreated 96 well microtiter plates with clear flat bottom wells (Costar, Corning Life Sciences, Union City, CA) bacteria were otherwise prepared, mixed with antibiotics or liposomes, and incubated according to the standard MIC procedure. Each assay included a growth control (incubation of bacteria in MHB+) and a killing control (incubation of bacteria with effective antibiotic). In addition, liposomes and antibiotics were incubated in the absence of bacteria to control for sterility of the reagents and to obtain background fluorescence readings. At the end of the incubation period, 100 μL of 1.25 μM Syto9 (diluted in dH_2_O) was added to each well. After 15 min incubation at RT in the dark, relative fluorescence units (RFU) were measured (485 nm_ex_/530 nm_em_; Tecan GENios, Tecan Systems, Inc., San Jose, CA). RFUs obtained from liposomal formulations or antibiotic incubated in medium without bacteria (background fluorescence) were subtracted from the RFUs obtained from corresponding wells with bacteria present. All liposomal formulations were first screened at 64 μg/mL test lipid. Those formulations that, compared to untreated bacteria, effected at least 95 % reduction of RFUs of at least two different bacterial strains were further investigated. They were subjected to serial dilution and determination of modified MIC. Modified MIC was defined as the lowest concentration of liposomal formulation that produced RFUs at background level.

### Determination of minimal bactericidal concentration (MBC)

After completion of the MIC assay, 6 µL of each well was serially diluted and spot plated. Based on Clinical and Laboratory Standards Institute guidelines M26-A [23], concentrations of liposomal formulations that reduced growth to less than 15 colony forming units (CFU)/6 μL or 2500 CFU/mL were considered bactericidal.

### Vancomycin synergism

The protocol for the modified MIC assay was employed with the following changes: instead of 10 μL of tenfold concentrated antibiotic or test lipid, 5 µL of 20-fold concentrated antibiotic and 5 µL of 20-fold concentrated test lipid was used. First, the MIC of vancomycin applied alone was established for VRE and then, serially diluted vancomycin was tested in the presence and absence of 5 μg/mL cholesteryl linoleate in carrier 3.

### Hemolytic activity testing

Sheep red blood cells (RBCs) at 50 % *v/v* whole blood in Alsever’s solution (Sigma-Aldrich) were washed by repeated centrifugation at 500×*g* and resuspension in saline (0.9 % *w/v*) until the absorbance of supernatant at 405 nm equaled the absorbance of saline. On the final wash, RBCs were adjusted to 15 % packed cell volume in normal saline (15 parts sedimented RBC plus 85 parts saline). In microplates, 10 μL of this was then added to 90 μL liposomal formulations or carrier which had been diluted in saline to the desired test concentration. As negative control, RBCs were mixed with saline. To obtain maximal (100 %) hemolysis, Triton X-100 (Fisher Scientific) was added to 1 % final concentration. Samples were then incubated at 37 °C, 7.5 % CO_2_ for up to 24 h. At the end of each incubation period (30 min, 3 h, and 24 h), the percent hemolysis was estimated by the quantification of liberated hemoglobin by absorbance reading. To prevent errors due to liposomal components affecting the oxidation state of the hemoglobin (and thus the absorbance reading) and to dissociate all major hemoglobins from other sample components (also affecting the absorbance reading), all hemoglobin species were converted to hematin based on the Alkaline Hematin Detergent—575 method [24]. Briefly, intact cells were pelleted by centrifugation at 4500×*g* and the resulting supernatants were diluted to 50 % *v/v* in 1 % Triton-X-100/0.5 M NaOH. Absorbance at 405 nm (Soret band) was measured in a Multiskan Ascent plate reader. After blanking with 50 % saline *v/v* in 1 % Triton X-100/0.5 M NaOH, the percent hemolysis was calculated, as follows: % H = (absorbance of treatment sample/absorbance of 100 % hemolysis sample) × 100.

### Cytotoxicity testing

Cytotoxic activity of liposomal formulations was assessed with a hepatic cell line that is commonly used for this purpose [25]. HepG2 cells (ATCC, Manassas, VA) were maintained in Dulbecco’s Modified Eagle Medium (DMEM) supplemented with 10 % fetal bovine serum, 1000 IU/mL penicillin, 100 μg/mL streptomycin and 2 mM l-glutamine (Invitrogen, Carlsbad, CA) in tissue culture flasks at 37 °C with 5 % CO_2_. Assays were performed in 96-well flat bottom tissue culture treated plates (Corning, Cambridge, MA). For passing, cells grown to 80 % confluency were detached with 0.25 % trypsin/0.53 mM EDTA (Cellgro, Mediatech, Inc, Manassas, VA) for 3 min, centrifuged at 1000×*g* for 8 min at 23 °C, and after resuspension in culture medium split at a ratio of 1: 4. For experiment seeding, cells were detached and spun as above, adjusted to 5 × 10^5^ viable cells/mL, and aliquoted at 100 μL per well. After incubation for 18–24 h, until 80 % confluency had been reached, supernatants were removed and replaced with 100 μL of liposome treatment (liposomal formulations in 9 % sucrose at 1000 μg/mL test lipid further diluted 1:10 in culture medium to yield a final concentration of 100 μg/mL test lipid). Stocks for carrier liposomes were diluted so that their phospholipid concentration was the same as the phospholipid concentration of the test lipid in its respective formulation. As negative control, 9 % sucrose diluted 1:10 in culture medium was used. After 48 h incubation at 37 °C with 5 % CO_2_ cell viability was determined with the XTT assay (Roche Diagnostics Pleasanton, CA) according to the manufacturer’s instructions. Previously untreated cells that were incubated for 15 min with 0.1 % Triton X 100 immediately prior to the XTT assay were used as positive control for cytotoxicity. Cytotoxic activity of lipids was quantified based on the inhibition of cell proliferation after lipid treatment relative to untreated cells.

### Data analysis

MIC and MBC assays were conducted in duplicates and repeated at least once. Hemolysis assays were performed in triplicate and cytotoxicity assays were conducted in replicates of four and repeated for a total of three independent experiments. Microsoft Excel 2013 was used for raw data analysis and graphing. IBM SPSS Statistics version 20 was used for statistical analysis.

## Results

### Validation of modified MIC assay with fluorescence readout

Due to their hydrophobicity- in particular cholesteryl esters cannot be dissolved in aqueous media- test lipids were incorporated into carrier liposomes. Six different formulations designated as carrier 1–6 were tested. The particulate nature of liposomes interferes with turbidity readings which are routinely used in standard MIC assays to evaluate bacterial growth in the presence and absence of antibiotics. Therefore, in this study, we employed a modified antimicrobial susceptibility testing assay that utilizes the green fluorescent DNA probe Syto9 to quantify bacterial proliferation [16]. As summarized in Table 2, the modified assay with fluorescence readout produces MICs that were mostly comparable to those determined with standard MIC testing, with both sets of MIC values within acceptable ranges as defined by the Clinical and Laboratory Standards Institute.

**Table 2:** Validation of the modified antimicrobial susceptibility testing assay employing the DNA binding fluoroprobe Syto9

Strains^a^	Antibiotic	MIC (μg/mL)^b^		
		Expected^c^	Measured	
			Standard assay	Modified assay
*S. aureus* ATCC 29213	Oxacillin	0.12–0.5	0.0625	0.5
	Cefotaxime	1–4	1–2	2
	Tetracycline	0.12–1	0.125–0.25	0.5
	Ciprofloxacin	0.12–0.5	0.25–0.5	0.5
*E. faecalis* ATCC 29212	Oxacillin	8–32	0.5–2	8–16
	Tetracycline	8–32	16	16
	Ciprofloxacin	0.25–1	0.5–1	2
*E. coli* ATCC 25922	Cefotaxime	0.03–0.12	0.125	0.125
	Tetracycline	0.5–2	1	2–4
	Ciprofloxacin	0.004–0.015	0.0156	0.0156
*P. aeruginosa* ATCC 27853	Cefotaxime	8–32	8	16
	Tetracycline	8–32	16	16–32
	Ciprofloxacin	0.25–1	0.25	0.5–1

^a^Designated ATCC quality control strains for antimicrobial susceptibility testing

^b^Shown are the values derived from two independent experiments each conducted in triplicate

^c^According to Clinical and Laboratory Standards Institute M100-S22 for broth microdilution assay

### Spectrum of activity of liposomal formulations

First, all formulations were screened at 64 μg/mL test lipid (free fatty acids or cholesteryl esters) against bacterial strains that cause HAI and are resistant to multiple antibiotics (Table 3). For comparison, a *P. aeruginosa* strain previously reported to be susceptible to cholesteryl esters (PACF, [16]) was also used. When test lipids were serially diluted, the corresponding carrier lipids were similarly diluted maintaining the ratio of test lipid to carrier lipid. For each test lipid the corresponding carrier formulation itself was also assayed at the equivalent concentration. Of note, the liposome size was influenced by the lipid species (see Additional file 1: Table S1).

**Table 3:** Screening of liposomal formulations for antibacterial activity

ID	SE		MRSA		VRE		ENC		PACF		PA		AB	
	TL	CR	TL	CR	TL	CR	TL	CR	TL	CR	TL	CR	TL	CR
PA1	64	−11	62	75	53	−89	−14	−80	4	32	20	13	23	−93
PA3	67	3	69	*82*	***99***	−63	14	−56	*84*	*92*	28	5	0	−91
PA4	***100***	8	32	62	***99***	−90	−10	6	3	15	41	45	35	−59
PA6	66	−11	42	59	65	−85	7	−48	12	34	−17	−22	46	−58
SA1	15	−14	63	63	−76	−91	22	−72	0	10	−7	18	28	−76
SA3	−28	−8	64	72	*83*	−78	22	−93	70	*93*	27	7	26	−60
SA4	***100***	7	38	64	***100***	−99	−18	−11	12	19	44	47	31	−63
SA6	5	−61	46	50	−25	−86	−30	−143	−16	27	−22	−25	29	−81
OA1	32	−21	62	79	40	−142	56	−60	22	38	−1	8	17	−77
OA3	50	−49	*80*	*83*	50	−41	−66	−117	*87*	*93*	−9	4	14	−92
OA4	31	−26	59	79	55	−58	−7	−25	12	17	52	45	31	−84
OA6	−10	0	66	59	−12	−18	44	30	11	25	−25	−21	1	−72
LA1	5	−27	49	70	25	−156	−31	−121	8	46	−6	12	18	−70
LA3	***100***	−57	55	*83*	18	−62	−37	−399	***97***	*93*	12	1	29	−61
LA4	*86*	−32	60	79	*94*	−101	*89*	−103	10	20	5	45	0	−75
LA6	9	−37	45	57	45	−156	−140	−135	2	25	−34	−26	22	−81
AA1	−37	51	48	73	66	−14	79	34	−1	44	−5	6	31	−85
AA3	72	*94*	50	*82*	−8	−40	−6	8	*91*	*94*	−17	−8	34	−57
AA4	65	36	*85*	*84*	*89*	43	*95*	54	15	18	51	47	33	−55
AA6	45	−69	45	58	33	−91	−1	−131	−8	14	−29	−20	34	−55
DA1	22	2	72	−42	−10	−11	34	42	38	−90	19	−91	−1	−76
DA3	−34	42	55	*95*	***98***	−105	35	15	71	*94*	−9	10	24	−77
DA4	15	1	28	54	−29	44	11	*80*	18	36	14	30	5	−67
DA6	10	18	44	55	−11	−40	−13	−14	−18	28	−32	−13	9	−71
CP1	*85*	***99***	74	74	−14	−7	36	70	27	43	−10	−19	21	−83
CP2	71	***100***	67	74	−15	3	15	9	*87*	66	−5	−6	15	−90
CP3	77	***99***	42	54	−44	5	5	39	*86*	*86*	24	28	4	−87
CP5	62	***100***	65	71	−6	7	9	58	*82*	63	−19	−9	15	−75
CO1	19	43	53	57	−11	−26	29	24	34	20	−9	−5	24	−69
CO3	10	***100***	59	70	62	−6	−34	−22	***99***	*95*	−25	−18	4	−78
CO4	−20	14	38	*90*	*84*	16	29	29	12	18	11	50	35	−56
CL1	NT	NT	64	67	NT	NT	NT	NT	*95*	72	−41	−26	NT	NT
CL3	***100***	52	65	47	21	−39	*94*	36	***100***	*88*	45	23	16	−82
CL4	NT	NT	*80*	68	NT	NT	NT	NT	*84*	33	−22	0	NT	NT
CA1	74	10	59	54	−16	−27	45	29	*82*	24	11	−4	19	−70
CA3	−44	11	60	65	11	−43	50	19	***100***	*93*	−20	−24	24	−72
CA4	53	*94*	53	*87*	31	***100***	47	−1	72	25	8	42	13	−68

Test lipids (see Table 1 for abbreviations) prepared in carrier liposomes formulations #1–6 with unique phospholipid composition (TL) and carrier liposomes without test lipid (CR) were screened against bacterial strains relevant to healthcare associated infections (*SE* multidrug-resistant *Staphylococcus epidermidis* ATCC 700566, *MRSA* methicillin resistant *Staphylococcus aureus* ATCC 33591, *VRE* vancomycin resistant *Enterococcus faecalis* ATCC 700802, *ENC* *Enterococcus cloacae* ATCC 49141, *PA* *Pseudomonas aeruginosa* ATCC 9027, *PACF* *Pseudomonas aeruginosa,* cystic fibrosis isolate [16]*, AB Acinetobacter baumanii* ATCC 19606). All test lipids where employed at 64 μg/mL. The corresponding carrier lipid concentrations were about 10–12 times higher for fatty acid formulations and 4–5 times higher for cholesteryl ester formulations. Bacterial growth was quantified based on relative fluorescence units (RFU) of the DNA probe Syto 9. Italics 80–95 % growth inhibition; Bolded and in italics >95 % growth inhibition. Data represent means of the % inhibition compared to untreated bacteria from two independent experiments conducted each in triplicate. *ID* Liposome identification, for example PA1 stands for palmitic acid prepared in carrier liposomes # 1

*NT* not tested

Based on an at least 80 % inhibition threshold, overall, test liposomes were more effective against the Gram-positive strains tested than the Gram-negative strains. Among the Gram-positive bacteria tested, *S. epidermidis* and VRE were inhibited by several formulations representing both fatty acids, in particular PA4 and SA4, and cholesteryl esters (CL3 and CA4 for *S. epidermidis* and VRE, respectively). Among the Gram-negative bacteria tested, one of the two *P. aeruginosa* strains and the *A. baumannii* strain were completely resistant to all lipids tested. Furthermore, the *E. cloacae* strain was inhibited only by two fatty acid formulations (LA4, AA4) and one cholesteryl ester formulation (CL3). However, the cystic fibrosis isolate of *P. aeruginosa* exhibited broad susceptibility to several fatty acid and cholesteryl ester formulations suggesting that there is a strain dependent susceptibility. In general, test lipids prepared with carrier 3 and carrier 4 demonstrated the greatest activity. There was also some inhibitory activity of the carrier lipids independent from the presence of fatty acids or cholesteryl esters (see Table 3).

Those test lipid formulations that effected at least 95 % growth inhibition against at least two different bacterial strains in the screening assay were further subjected to determination of minimal inhibitory concentration (Table 4). The lowest MIC values were determined for formulations PA4, SA4, and CL3 for *S. epidermidis* and VRE. The MIC for PA4 was 0.5 μg/mL against *S. epidermidis* and 2 μg/mL against VRE. The MIC for SA4 was 0.25 μg/mL against *S. epidermidis* and 0.5 μg/mL against VRE. For CL3 the MIC was 1 μg/mL against *S. epidermidis* and 8 μg/mL against *PACF.* The activity of LA3 against PACF in the screening assay was not confirmed by the MIC assay.

**Table 4:** Minimal inhibitory concentrations (MIC, given in μg/mL) of test lipids that effected a growth inhibition of >95 % against at least two different bacterial strains in the screening assay

Liposomal formulation^a^	Bacterial strain^b^		
	*SE*	*VRE*	*PACF*
PA4	0.50	2	NT
SA4	0.25	0.50	NT
LA3	64	NT	>64
CL3	1	NT	8

Shown are MIC values derived from two independent experiments each performed in triplicate. *NT* not tested because not susceptible in the screening assay

^a^See Table 1 for letter abbreviations; numbers indicate the type of liposomal formulation used

^b^ *SE* multidrug-resistant *Staphylococcus epidermidis* ATCC 700566, *VRE* vancomycin resistant *Enterococcus faecalis* ATCC 700802, *PACF* *Pseudomonas aeruginosa*, cystic fibrosis isolate

### Bactericidal activity of selected liposomal formulations

**Fig. 1:**
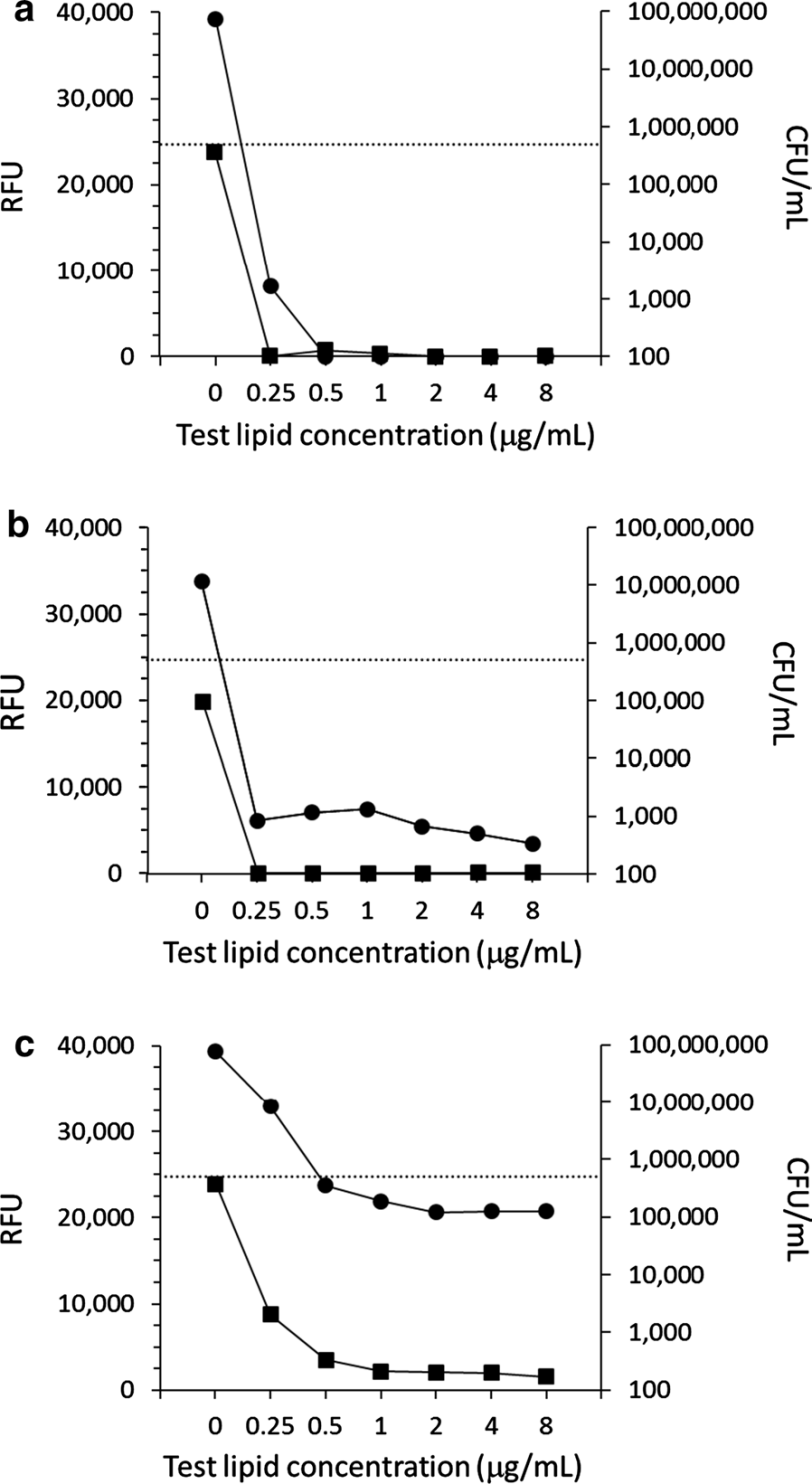
Minimal bactericidal concentration of liposomal formulations in comparison with their minimal inhibitory concentration. Modified minimal inhibitory concentration (MIC) assay (*square symbols*, *left* *y-axis*) was performed with *S. epidermidis* followed by determination of minimal bactericidal concentration (*round symbols*, *right y-axis*). Test lipids were **a** palmitic acid prepared in carrier-4 (PA4); **b** stearic acid prepared in carrier 4 (SA4); and **c** cholesteryl linoleate prepared in carrier-3 (CL3). *RFU* relative fluorescence units. *Dotted line* bacterial inoculum (CFU/mL). Shown are means of two experiments conducted in duplicates

The liposomal formulations PA4, SA4 and CL3 were then subjected to testing for minimal bactericidal concentration (MBC) against *S. epidermidis* (Fig. 1). PA4 and SA4 demonstrated strong bactericidal activity (MBC of 0.5 and 0.25 μg/mL, respectively). In contrast, CL3 was not bactericidal at the tested concentrations, reducing the bacterial inoculum by no more than 75 %.

### Effect of liposomes on vancomycin susceptibility

**Fig. 2:**
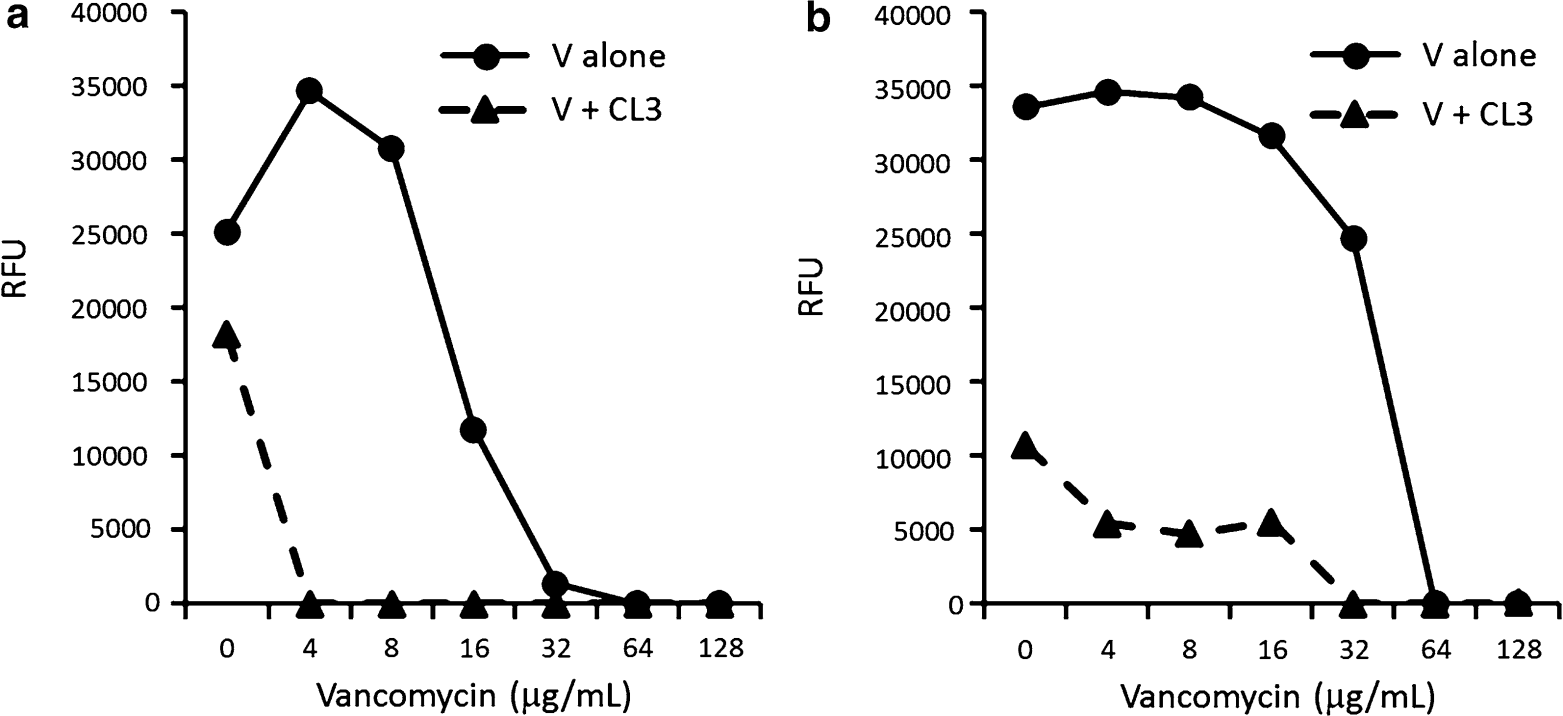
Minimal inhibitory concentration (MIC) of liposomal cholesteryl linoleate combined with standard antibiotic. Modified MIC assay with Syto 9 fluorescence read out for vancomycin-resistant *Enterococcus faecalis* incubated with vancomycin (V) in the presence and absence of cholesteryl linoleate prepared in carrier-3 (CL3, 5 μg/mL). *RFU* relative fluorescence units. Shown are averages of triplicate measurements of two independent experiments (**a**, **b**)

To address whether antimicrobial lipids in liposomal formulation may have the potential to augment the effects of standard antibiotics, MIC of vancomycin was determined for VRE in the presence and absence of CL3, which alone—or its carrier liposomes—did not effect a substantial growth inhibition (see Table 3), or carrier liposomes. The MIC of vancomycin against VRE shifted from 64–32 μg/mL to as low as 4 μg/mL in the presence of 5 μg/mL CL3 (Fig. 2), which would be consistent with a shift from vancomycin resistant to susceptible, according to CLSI-M100-S22. This suggests that liposomal lipids may potentiate or restore the efficacy of antibiotics against multidrug-resistant bacteria.

### Hemolytic and cytotoxic activity of selected liposomal formulations

**Fig. 3:**
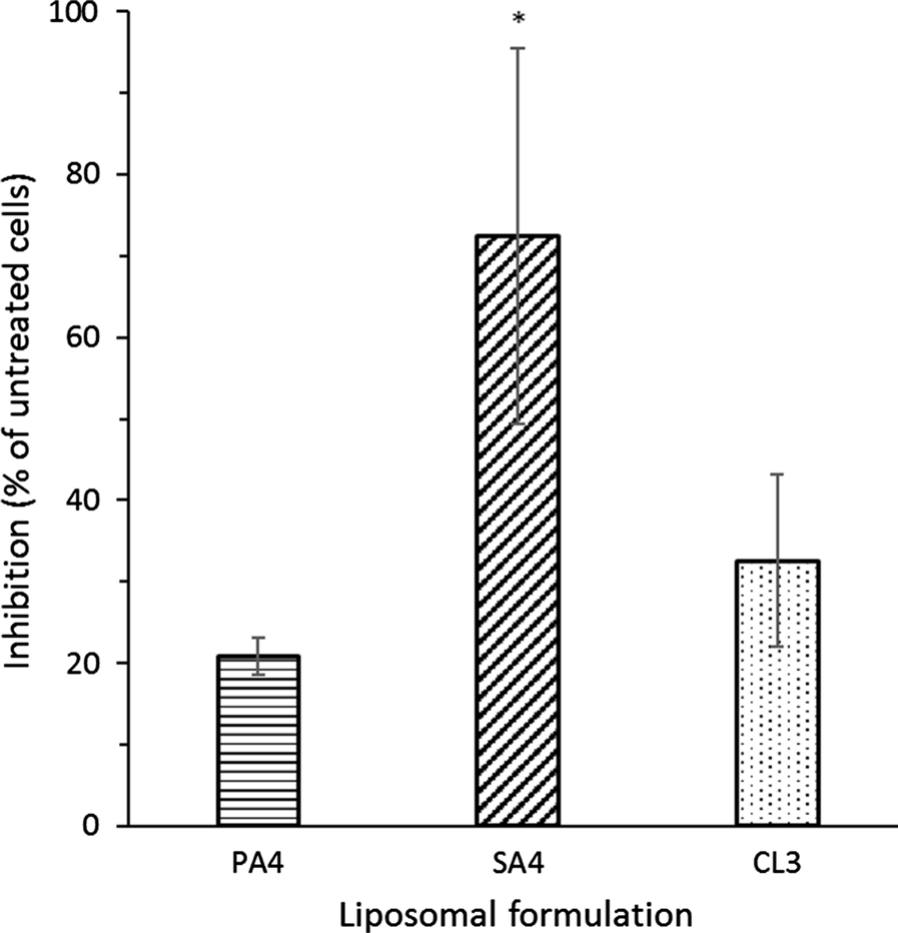
Cytotoxic activity of the most potent antimicrobial lipids against the cell line HepG2. Metabolically active cells were quantified after 48 h incubation in the presence and absence of 100 μg/mL test lipid using the XTT assay. *PA4* palmitic acid prepared in carrier-4; *SA4* stearic acid prepared in carrier-4; *CL3* cholesteryl linoleate prepared in carrier-3. Shown are mean ± S.D., n = 3. *p = 0.001 in one-way ANOVA with Bonferroni posthoc analysis

Prior to considering clinical use, a candidate compound must be evaluated for hemolytic and cytotoxic activity. PA4, SA4, and CL3 were subjected to hemolysis testing (Table 5) and cytotoxicity testing (Fig. 3). At high dose (900 μg/mL test lipid) PA4 and SA4 effected over 40 % hemolysis within 30 min and CL3 within 3 h. However, when tested at 90 μg/mL test lipid (between 90 and 360 times the lowest observed MIC) none of the formulations exhibited significant hemolysis over 3 h. Carrier-3 and carrier-4, in the absence of test lipid, also exerted hemolytic effects at the higher dose (both over 40 % hemolysis after 3 h), but did not cause hemolysis when tested at 90 μg/mL over 3 h (2.24 ± 0.6 and 1.64 ± 0.2 % hemolysis, respectively).

**Table 5:** Hemolytic activity of the most potent antimicrobial lipids

Liposomal formulation	Hemolysis (% maximal hemolysis) at 900 μg/mL			At 90 μg/mL^a^		
	30 min	3 h	24 h	30 min	3 h	24 h
PA4	>40	>40	>40	1.61 ± 0.50	3.10 ± 1.00	70.43
SA4	>40	>40	>40	2.21 ± 1.80	4.04 ± 0.60	46.35
CL3	<20	>40	>40	1.13 ± 0.20	0.80 ± 0.10	54.55

Hemolysis was screened at 900 μg/mL lipid concentration and further tested at 90 μg/mL lipid concentration (mean ± S.D., n = 3 for 30 min and 3 h time points and n = 1 for 24 h time point). *PA4* palmitic acid prepared in carrier-4, *SA4* stearic acid prepared in carrier-4, *CL3* cholesteryl linoleate prepared in carrier-3

^a^There were no statistically significant differences compared to control erythrocytes after 30 min and 3 h

Cytotoxic activity was evaluated with HepG2 cells at 100 μg/mL employing the metabolic XTT assay (Fig. 3). While SA4 had significant inhibitory effects, cells treated with PA4 and CL3 were statistically not different from untreated cells. Carrier-3 and carrier-4, when tested alone, also exhibited cytotoxic effects (40.68 ± 12.54 % inhibition, p < 0.05, and 83.97 ± 13.88 % inhibition, p < 0.001, respectively). This data suggests that the hemolytic and cytotoxic activity of the liposomal formulations is at least in part contributed by the carrier lipid. Considering the varying activities of different lipids incorporated in the same carriers, a complex interaction between the different lipid species is likely.

## Discussion

In this study, striving to address the urgent public health need posed by HAI with multidrug-resistant bacteria, we assessed the potential of free fatty acids and cholesteryl esters as novel antibacterial agents. Liposomes of varying composition were employed as carrier for these lipophilic agents and these formulations were tested against Gram-positive and Gram-negative multidrug-resistant bacteria involved in HAI using a modified MIC. The potential for restoring activity of conventional antibiotics was probed and hemolytic and cytotoxic activity assayed. Palmitic and stearic acid as well as cholesteryl linoleate were identified as the most promising compounds whereby the carrier liposome formulation influenced their activity.

To overcome interference of turbidity contributed by liposomes we employed the DNA probe Syto9 to quantify bacterial growth in an otherwise standard MIC assay. Styo9 has been often used to quantify viable bacteria in biofilms [26, 27] but not yet been validated for use in MIC assays, which we did here using quality control strains and standard antibiotics. The modified DNA probe-based MIC assay may also be useful for testing fastidious organisms that require the addition of blood products in their growth media making turbidity based measurements including standard MIC testing obsolete.

Among the fatty acids and cholesteryl esters tested, palmitic acid, stearic acid, and cholesteryl linoleate emerged as the most active lipids. Palmitic acid and stearic acid acted only against multidrug-resistant *S. epidermidis* and vancomycin resistant *E. faecalis,* both representing Gram-positive cocci. This is in line with an earlier report on the preferential bactericidal activity of fatty acids against Gram-positive bacteria [28] though the stated effective concentrations of palmitic acid were substantially higher than what we describe here. The relatively decreased activity reported by Huang et al. [28] may be attributed to the use of ethanol-solubilized fatty acid without a carrier underlining the potential of liposomal formulations to reduce required active concentrations. Cholesteryl linoleate showed activity against the multidrug-resistant *S. epidermidis* and a *P. aeruginosa* strain but was less effective in killing than stearic acid and palmitic acid. This suggests that the cholesteryl ester may have a different mode of action.

We have identified host derived lipids that may have promise for future use as antimicrobials when applied alone or in combination with standard antibiotics. Plant lipids, namely essential oils, have long been used for their antibacterial activity in the food industry [29, 30]. Recently, though, they have been also assessed for clinical use [31, 32], including the use as enhancer of antibiotics [33]. In a rabbit sepsis model, co-administration of the polyunsaturated fatty acids linolenic and arachidonic acid with antibiotics enhanced killing of a multidrug-resistant *P. aeruginosa* strain [34]. Adding lipophilic properties to antimicrobial peptides by N-acylation has also been proven to enhance their antimicrobial activity [35, 36].

We observed that the carrier liposome composition influenced the test lipid activity and that some formulation exhibited independent antibacterial activity. This suggests that phospholipid-mediated antimicrobial activity could contribute to the low MIC of palmitic acid and stearic acid. An interaction between the carrier lipids and the test lipids is also implicated by the changes in liposomes size depending on the constituents. Possible mechanisms of the observed liposome activity may include fusion and subsequent disruption of the bacterial cell membrane through lipid embedding into the phospholipid bilayer and, for unsaturated lipids, lipid peroxidation with radical production [15]. Such mechanistic studies are currently under way in our laboratory.

Bacterial and mammalian cell membranes both are composed of phospholipid bilayers. Thus, it is conceivable that the antibacterial liposome formulations exhibit hemolytic and cytotoxic activity against mammalian cells. At concentrations ninety times or higher than the lowest MIC no hemolytic activity was observed within the first 3 h but prolonged incubation resulted in hemolysis. This suggests that topical use may be a preferable treatment strategy for liposomal lipids. Cytotoxic effects through lipid peroxidation and mitochondria-initiated apoptosis have been well documented, in particular for unsaturated fatty acids such as linoleic acid [37, 38], and cytotoxic fatty acids are under investigation for intended use in cancer treatment [39, 40]. Here, we found that palmitic acid and cholesteryl linoleate did not cause significant growth inhibition of HepG2 cells but that stearic acid displayed significant cytotoxicity. Considering that the degree of antibacterial activity was influenced by the carrier composition and that the carriers also exerted some hemolytic and cytotoxic effects, careful selection and modification of the carrier lipids may yield antimicrobial formulations devoid of hemolytic and cytotoxic activity.

Liposomes have been previously used as carrier for antimicrobial compounds. For example, the antifungal drug amphotericin B was successfully formulated in liposomes with greatly reduced cytotoxicity [41]. Other applications include liposomal delivery of antibiotics to pathogens entrapped in biofilms and intracellular pathogens [42, 43]. A major advantage of using liposomes as carrier for antimicrobial lipids (and other compounds) is the possibility to insert molecules that enable targeted delivery of the drug [44].

## Conclusions

In summary, our results showed that selected fatty acids and cholesteryl esters packaged with phospholipids exhibit antibacterial activity against Gram-positive and Gram-negative bacterial species and may augment the activity of antibiotics. Bactericidal activity could be unlinked from hemolytic and cytotoxic activity. The type of phospholipid carrier greatly influenced the activity. Thus, fatty acids and cholesteryl esters may have potential as novel lipophilic antimicrobial agents whereby modulating the phospholipid composition and incorporating tags may enable targeting specific types of bacteria.

## Additional file

10.1186/s13104-016-2138-8 Test lipid composition and particle size.

## Abbreviations

CFU: colony forming units
CL: cholesteryl linoleate
HAI: healthcare associated infections
MBC: minimal bactericidal concentration
MDR: multidrug resistant
MIC: minimal inhibitory concentration
PA: palmitic acid
RBC: red blood cell
RFU: relative fluorescence unit
SA: stearic acid
XTT: 2,3-Bis-(2-Methoxy-4-Nitro-5-Sulfophenyl)-2H-Tetrazolium-5-Carboxanilide
