# Supplementary material for: Assessment of free fatty acids and cholesteryl esters delivered in liposomes as novel class of antibiotic
Source: BMC Res Notes. 2016 Jul 8;9:337. doi: 10.1186/s13104-016-2138-8 (PMC4938966; doi:10.1186/s13104-016-2138-8)
Supplement: Supplementary file 1 — 10.1186/s13104-016-2138-8 Test lipid composition and particle size. [file 13104_2016_2138_MOESM1_ESM.pdf]

Cheung *et al*., Additional File 1: Test lipid composition and particle size

Test Lipid		I.D.	Carrier	[Test Lipid] mg/ml	[Total Lipid] mg/ml	[Carrier Lipid] mg/ml	Size nm
Carrier	None	037-18A	Carrier A	0.00	15	15.00	58
Carrier	None	037-09B	Carrier B	0.00	10	10.00	NA
Carrier	None	037-18C	Carrier C	0.00	15	15.00	48
Carrier	None	037-18D	Carrier D	0.00	15	15.00	15
Carrier	None	037-09E	Carrier E	0.00	10	10.00	NA
Carrier	None	037-18F	Carrier F	0.00	15	15.00	93
Palmitic acid	PA1	037-19I1	Carrier A	1.14	15	13.86	47
Palmitic acid	PA3	037-16I2	Carrier C	0.86	10	9.14	NA
Palmitic acid	PA4	037-16I3	Carrier D	1.13	15	13.87	49
Palmitic acid	PA6	037-16I4	Carrier F	0.61	7.5	6.89	67
Palmitic acid	SA1	037-17J1	Carrier A	0.83	10	9.17	3246
Stearic acid	SA3	037-17J2	Carrier C	0.95	10	9.05	553
Stearic acid	SA4	037-17J3	Carrier D	0.83	10	9.17	25
Stearic acid	SA6	037-17J4	Carrier F	0.45	5	4.55	187
Oleic acid	OA1	037-16H1	Carrier A	0.83	10	9.17	31
Oleic acid	OA3	037-16H2	Carrier C	0.94	10	9.06	29
Oleic acid	OA4	037-16H3	Carrier D	0.83	10	9.17	13
Oleic acid	OA6	037-16H4	Carrier F	0.45	5	4.55	21
Linoleic acid	LA1	037-15G1	Carrier A	0.82	10	9.18	31
Linoleic acid	LA3	037-15G2	Carrier C	0.94	10	9.06	250
Linoleic acid	LA4	037-15G3	Carrier D	0.82	10	9.18	72
Linoleic acid	LA6	037-15G4	Carrier F	0.44	5	4.56	17
Arachidonic acid	AA1	037-15F1	Carrier A	0.89	10	9.11	155
Arachidonic acid	AA3	037-15F2	Carrier C	1.01	10	8.99	99
Arachidonic acid	AA4	037-15F3	Carrier D	0.88	10	9.12	69
Arachidonic acid	AA6	037-15F4	Carrier F	0.48	5	4.52	17
Docosahexaenoic acid	DA1	037-05B1	Carrier A	0.95	10	9.05	NA
Docosahexaenoic acid	DA3	037-05B2	Carrier C	1.08	10	8.92	NA
Docosahexaenoic acid	DA4	037-05B3	Carrier D	0.95	10	9.05	NA
Docosahexaenoic acid	DA6	037-16B4	Carrier F	0.50	10	9.50	NA
Cholesteryl palmitate	CP1	037-07C1	Carrier A	0.83	5	4.17	NA
Cholesteryl palmitate	CP2	037-07C4	Carrier B	0.88	5	4.12	NA
Cholesteryl palmitate	CP3	037-07C2	Carrier C	0.94	5	4.06	NA
Cholesteryl palmitate	CP5	037-07C6	Carrier E	0.67	5	4.33	NA
Cholesteryl oleate	CO1	037-14E1	Carrier A	0.86	5	4.14	68
Cholesteryl oleate	CO3	037-14E2	Carrier C	0.97	5	4.03	37
Cholesteryl oleate	CO4	037-14E3	Carrier D	0.86	5	4.14	71
Cholesteryl linoleate	CL1	037-05A1	Carrier A	0.86	5	4.14	NA
Cholesteryl linoleate	CL3	037-05A2	Carrier C	0.97	5	4.03	NA
Cholesteryl linoleate	CL4	037-05A3	Carrier D	0.86	5	4.14	NA
Cholesteryl arachidonate	CA1	037-14D1	Carrier A	0.89	5	4.11	86
Cholesteryl arachidonate	CA3	037-14D2	Carrier C	0.99	5	4.01	59
Cholesteryl arachidonate	CA4	037-14D3	Carrier D	0.88	5	4.12	75

NA: data not available
